# Duvyzat (givinostat): a new hope for Duchenne muscular dystrophy patients

**DOI:** 10.1097/MS9.0000000000003196

**Published:** 2025-03-28

**Authors:** Nabiha Syed, Zaib Un Nisa Mughal, Abdul Haseeb, Gaffar Alemam Manhal, Abdullatif Yasir Eissa, Khabab Abbasher Hussien Mohamed Ahmed

**Affiliations:** a Department of Medicine, Dow University of Health Sciences, Karachi, Pakistan; b Department of Medicine, Jinnah Sindh Medical University, Karachi, Pakistan; c Faculty of Medicine, University of Khartoum, Khartoum, Sudan

**Keywords:** Duchenne muscular dystrophy (DMD), Duvyzat, givinostat, histone deacetylase inhibitors

## Abstract

The approval of Duvyzat (givinostat) by the Food and Drug Administration (FDA) marks a significant milestone in the treatment of Duchenne muscular dystrophy (DMD), offering renewed hope for patients and their families. DMD is a rare genetic disorder characterized by progressive muscle degeneration and weakness, often leading to loss of mobility and life-threatening complications. Currently, there is no cure for DMD, and available treatments focus on managing symptoms and slowing disease progression. Duvyzat belongs to a class of drugs known as histone deacetylase inhibitors, which have shown promise in preclinical studies for their ability to modulate gene expression and potentially improve muscle function in DMD. Clinical trials evaluating the safety and efficacy of Duvyzat in DMD patients have demonstrated encouraging results, prompting the FDA’s approval. The approval of Duvyzat represents a significant advancement in the field of DMD therapeutics, providing patients with a much-needed treatment option that targets the underlying mechanisms of the disease. By inhibiting histone deacetylases, Duvyzat has the potential to restore dystrophin expression, reduce inflammation, and preserve muscle integrity, ultimately improving the quality of life for individuals with DMD. However, it is essential to recognize that while Duvyzat represents a step forward, it is not a cure for DMD. Continued research and development efforts are needed to further refine treatment approaches and address the complex challenges associated with this devastating disorder. Nonetheless, the approval of Duvyzat offers hope and optimism for the DMD community, signaling progress toward more effective therapies and improved outcomes for patients.

## Introduction

Duchenne muscular dystrophy is a severe, progressive, muscle-wasting condition that leads to movement-related challenges, and eventually results in requirement for assisted ventilation and early death. The illness is due to mutations in the Duchenne muscular dystrophy (DMD) gene, which prevents muscles from producing dystrophin, a crucial protein that links the cytoskeleton of muscle cells to the dystrophin-glycoprotein complex (DGC). Dystrophin stabilizes the muscle cell membrane during contraction and prevents damage from mechanical stress. It also plays a role in the cellular signaling that promotes muscle repair. Associated proteins like sarcoglycans and dystroglycan are integral to maintaining membrane integrity and linking dystrophin to the extracellular matrix. The absence of dystrophin disrupts this complex, resulting in membrane instability, cell necrosis and an inflammatory response that leads to progressive muscle degeneration and replacement with fibrotic tissue, ultimately causing severe muscle weakness. Muscles lacking dystrophin are more vulnerable to injury, which leads to progressive loss of muscle mass and function, and presentations such as cardiomyopathy. Understanding these mechanisms is vital for developing targeted therapies for DMD.

The incidence of DMD is estimated to be 1 in 5000 live male births, worldwide. Patients first exhibit symptoms at 2–3 years of age, which include trouble climbing stairs, a waddling gait and frequent falls. Around ages of 10–12, most patients become dependent on wheelchairs, and by the time they are 20, they require assisted ventilation. The majority of DMD patients pass away from heart or respiratory failure between the ages of 20 and 40 while receiving the best care possible^[1,2]^.

It is important to adhere to the guidelines for genetic diagnosis of DMD. Speech delay and cognitive impairment are present in 30% of patients with DMD. When young boys (2–4 years old) exhibit hypertrophic calves, delayed motor milestones, muscle weakness and the Gowers’ sign, DMD should be suspected, and plasma creatine kinase (CK) levels should be measured; patients with DMD typically have significantly elevated CK levels from birth, with values sometimes higher than 20 000 U/L. In addition, because of the continuous muscle degeneration, patients with DMD also have elevated plasma levels of aspartate transaminase (AST) and alanine transaminase (ALT) levels. However, the routine diagnostic work-up for DMD doesn’t include ALT and AST level assessment^[1,3]^.

The field of therapeutic options for DMD has advanced significantly in clinical trials over the past ten years, with two therapeutic approaches identified: (1) gene therapy and (2) attempts to reverse or block the disease’s pathophysiological processes such as inflammation, fibrosis and muscle regeneration. Currently, although there is no viable treatment for the declining muscle function in DMD, there are few treatment methods that are used in managing the illness. Gene transfer, utilizing the safe, non-pathogenic adeno-associated-virus vector to restore dystrophin expression is one of the promising treatment approaches for this fatal illness. It has demonstrated remarkable treatment efficacy in big animal models for DMD. Corticosteroids, which are now recommended in early stages of disease and are beneficial for DMD, are the other medication that has been demonstrated to alter natural history of disease independent of genetic abnormalities. Using synthetic phosphorodiamidate morpholino oligomers, a subclass of antisense oligonucleotides that bind to pre-mRNA, is another possibility for pharmacological treatment; it modifies the splicing process and skips the targeted exon.

The lack of the Food and Drug Administration (FDA)-approved drugs specifically for DMD underscored critical need for innovative treatment agents and non-invasive diagnostic techniques^[4,5]^. An important development in neurology is the FDA-approved oral suspension Duvyzat (givinostat), for DMD in patients of 6 years of age or older. Duvyzat is a histone deacetylase (HDAC) inhibitor regulates the dystrophic muscle’s dysregulated HDAC activity, which is a major component of the dystrophin deficiency associated with DMD. By blocking HDAC pathogenic overactivity, Duvyzat’s mechanism of action seems to be able to counteract the disease pathology and slow down the degradation of muscles by addressing the series of events that contribute to muscle damage. One of the first deficits in DMD is mitochondrial dysfunction, which results in less than 50% of ATP content in dystrophic muscles and is caused by variety of cellular stresses. A study found that there are there are two temporally distinct phases of mitochondrial damage, with early stages characterized by decrease in mitochondrial mass and later stages an accumulation of dysfunctional mitochondria. This results in different patterns of oxidative fibers and progressive impairment of mitochondrial biogenesis linked to increased deacetylation of promoter of peroxisome proliferator-activated receptor-gamma coactivator 1**α (**PGC-1**α)**. Givinostat inhibits this kind of histone deacetylation, which positively changes the PGC-1**α** promoter’s epigenetic profile and supports mitochondrial biogenesis and oxidative fibers^[6]^.

Due to its distinct mode of action, givinostat has potential to be therapeutic for DMD. However, there are certain negative effects associated with the use of givinostat. The most frequent adverse effects are nausea, vomiting, diarrhea and abdominal pain. Givinostat’s inhibition of HDACs can lead to altered gene expression in the gastrointestinal tract. This may disrupt normal gut motility, secretory functions, and mucosal integrity, resulting in above mentioned symptoms. Givinostat can influence platelet production and lifespan. By modulating the expression of genes involved in thrombopoiesis and platelet function, it may lead to reduced platelet counts. HDAC inhibition can also affect megakaryocyte differentiation in the bone marrow, potentially resulting in thrombocytopenia. It is possible to experience musculoskeletal symptoms like myalgia and arthralgia in addition to dermatological reactions like rash and itching. Abnormalities in liver function, increased transaminases and raised triglycerides may arise, requiring close observation throughout the course of treatment. There have been instances of cardiac damage linked to QT interval prolongation. When using givinostat, patients should be constantly watched for these possible adverse effects and health care professionals should carefully consider the advantages against the risks before initiating treatment^[7]^. This medication is taken orally twice daily with food and the suggested dosage is based on body weight, age, co-morbidities, baseline platelet counts and triglycerides. If the patient has moderate to severe diarrhea, platelet count that is less than 150 × 109/L and fasting triglycerides of more than 300 mg/dl, the indicated and calculated dosage is reduced^[7,8]^.

Givinostat’s efficacy in treating Duchenne muscular dystrophy was assessed throughout the course of an 18-month randomized, double-blind, placebo-controlled trial. A total of 179 patients were randomly assigned to either a placebo (n = 61) or givinostat (n = 118) in 2:1 ratio. A dosage schedule based on weight was used. Male patients aged 6 years and above with verified diagnosis of DMD who were ambulatory and taking consistent dose of corticosteroids for a minimum of 6 months were included in the trial. Patients were excluded from the study if they had the following abnormalities at the screening visit: platelet, white blood cell, or hemoglobin counts less than the lower limit of normal, triglycerides >300 mg/dL (3.42 mmol/L) in fasting condition, or had a baseline-corrected QT interval, Fridericia’s correction (QTcF) of >450 msec (mean of 3 consecutive readings 5 minutes apart) or a history of additional risk factors for torsades de pointes (e.g., heart failure, hypokalemia, or family history of long QT syndrome). Overall, 2% of the patients discontinued the study because of adverse reactions. The main outcome measure was the difference in 4-stair-climb (4SC) times for givinostat and placebo between baseline and month 18. The 4SC measures how long it takes to climb four stairs as a proxy for muscular function. The shift in the secondary efficacy goal from baseline to Month 18 according to North Star Ambulatory Assessment’s assessment of physical function (NSAA). Based on a predetermined range of baseline muscle fat fraction as established by MR spectroscopy, the primary analysis population was created. Compared to placebo, patients receiving givinostat had statistically significant reduction in deterioration in 4SC. Based on pre-specified multiplicity adjustment, patients treated with givinostat showed reduced worsening on NSAA when compared to placebo, which was nominally significant but not statistically significant.

The primary adverse effects observed in the givinostat group were vomiting and diarrhea, and treatment-related deaths were reported (see Table 1). During the trial, the four-stair climb evaluation findings among ambulant boys with DMD decreased in both groups, but the reduction was significantly less with givinostat than with placebo. Following a safety review, the dosage of givinostat was reduced, but no new safety signals were detected. Ongoing clinical trials are currently assessing the long-term safety and efficacy of givinostat.^[7]^

**Table 1:** Common adverse reactions reported in DUVYZAT-treated patients as compared to placebo

Adverse reactions	DUVYZAT	Placebo
	N = 118	N = 61
	%	%
Diarrhea	37	20
Abdominal pain	34	25
Thrombocytopenia	33	0
Nausea	32	18
Hypertriglyceridemia	23	7
Pyrexia	13	8
Myalgia	9	3
Rash	9	2
Arthralgia	8	2
Fatigue	8	0
Decrease appetite	7	0
Constipation	7	2

In conclusion, the study on givinostat highlights its promising potential as a therapeutic option for DMD. The drug has shown encouraging results in enhancing muscle function and reducing inflammation. However, to fully harness its benefits, future research should focus on combination therapies. By exploring the synergistic effects of givinostat with other existing treatments, such as corticosteroids or exon-skipping therapies, researchers could develop more effective strategies to improve patient outcomes.

Additionally, investigating the efficacy of givinostat across different subtypes of DMD will be crucial. DMD is a heterogeneous condition, and variations in genetic mutations may influence treatment responses. Conducting studies that assess how givinostat affects various subtypes could help tailor more personalized treatment approaches. Overall, expanding the research on givinostat could lead to significant advancements in the management of DMD, offering hope for better quality of life for affected individuals.

## Data Availability

None.
